# Molecular Mechanical Differences between Isoforms of Contractile Actin in the Presence of Isoforms of Smooth Muscle Tropomyosin

**DOI:** 10.1371/journal.pcbi.1003273

**Published:** 2013-10-24

**Authors:** Lennart Hilbert, Genevieve Bates, Horia N. Roman, Jenna L. Blumenthal, Nedjma B. Zitouni, Apolinary Sobieszek, Michael C. Mackey, Anne-Marie Lauzon

**Affiliations:** 1 Dept. Physiology, McGill University, Montréal, Québec, Canada; 2 Centre for Applied Mathematics in Bioscience and Medicine, Montréal, Québec, Canada; 3 Meakins-Christie Laboratories, McGill University, Montréal, Québec, Canada; 4 Dept. Biomedical Engineering, McGill University, Montréal, Québec, Canada; 5 Institute for Biomedical Aging Research, Austrian Academy of Sciences, Innsbruck, Austria; 6 Dept. Physics and Dept. Mathematics, McGill University, Montréal, Québec, Canada; 7 Dept. Medicine, McGill University, Montréal, Québec, Canada; University of California San Diego, United States of America

## Abstract

The proteins involved in smooth muscle's molecular contractile mechanism – the anti-parallel motion of actin and myosin filaments driven by myosin heads interacting with actin – are found as different isoforms. While their expression levels are altered in disease states, their relevance to the mechanical interaction of myosin with actin is not sufficiently understood. Here, we analyzed *in vitro* actin filament propulsion by smooth muscle myosin for 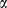-actin (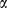
A), 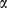-actin-tropomyosin-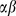 (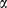
A-Tm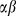), 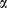-actin-tropomyosin-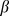 (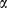
A-Tm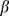), 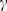-actin (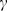
A), 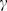-actin-tropomyosin-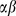 (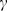
A-Tm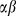), and 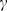-actin-tropomoysin-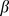 (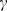
A-Tm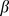). Actin sliding analysis with our specifically developed video analysis software followed by statistical assessment (Bootstrapped Principal Component Analysis) indicated that the *in vitro* motility of 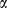
A, 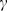
A, and 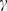
A-Tm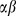 is not distinguishable. Compared to these three ‘baseline conditions’, statistically significant differences (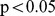) were: 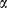
A-Tm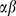 – actin sliding velocity increased 1.12-fold, 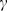
A-Tm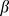 – motile fraction decreased to 0.96-fold, stop time elevated 1.6-fold, 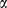
A-Tm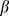 – run time elevated 1.7-fold. We constructed a mathematical model, simulated actin sliding data, and adjusted the kinetic parameters so as to mimic the experimentally observed differences: 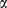
A-Tm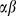 – myosin binding to actin, the main, and the secondary myosin power stroke are accelerated, 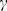
A-Tm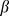 – mechanical coupling between myosins is stronger, 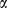
A-Tm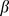 – the secondary power stroke is decelerated and mechanical coupling between myosins is weaker. In summary, our results explain the different regulatory effects that specific combinations of actin and smooth muscle tropomyosin have on smooth muscle actin-myosin interaction kinetics.

## Author Summary

Dependent on the required physiological function, smooth muscle executes relatively fast contraction-relaxation cycles or maintains long-term contraction. The proteins driving contraction – amongst them actin, tropomyosin, and the contraction-driving myosin motor – can show small changes in the way they are constructed, they can be expressed as different “isoforms”. The isoforms are supposedly tailored to support the specific contraction patterns, but for tropomyosin and actin it is unclear exactly how the isoforms' differences affect the interaction of actin and myosin that generates the muscle contraction. We measured actin movement outside the cellular environment, focusing on the effects of different isoform combinations of only actin, myosin, and tropomyosin. We found that the actin isoforms cause differences in the mechanical interaction only when tropomyosin is present, not without it. Also, all different actin-tropomyosin combinations affected the mechanical interactions in a different way. In our experiments we could not directly observe the mechanical interactions of actin, tropomyosin, and myosin, so we reconstructed them in a mathematical model. With this model, we could determine in detail how the different actin-tropomyosin combinations caused the differences that we observed in our experiments.

## Introduction

### Smooth muscle contractile protein expression

Differential expression of smooth muscle contractile proteins has been associated with organismal development [1], contractile phenotypes [2]–[4], and pathologies, e.g. preterm labour, hypertrophic bladder, or airway hyper-responsiveness [5]–[7]. While the role of the smooth muscle myosin isoforms has been extensively investigated [7]–[9], the functional implications of the differential expression of specific actin and actin-regulatory protein isoforms remain elusive [4].

### Smooth muscle actin

In smooth muscle, actin isoforms are expressed from four different genes, yielding “vascular muscle” 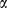
- and “enteric muscle” 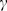-actin, as well as non-muscle (cytoplasmic) 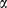
- and 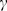-actin. The muscle isoforms are associated with the contractile apparatus, the non-muscle isoforms with cytoskeletal structures [5]. Muscle 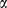-actin is generally associated with tonic, 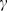-actin with phasic smooth muscles [5], [10], [11]. An anti-proportional relationship between the absolute levels of 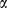
- and 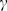-actin has been established [2]. Disease-related expression differences in 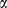
- vs. 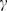-actin have been found [6]. Functional differences between 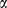
- and 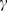-isoforms were searched for in molecular mechanics experiments, but, to our knowledge, no differences were detected [12]–[15]. Insight from tissue level mechanics seems lacking, too [4].

### Smooth muscle tropomyosin

Smooth muscle tropomyosin affects the weak to strong binding of ATP-activated myosin to actin: tropomyosin can be in an ON state supporting myosin strong binding, or an OFF state hindering myosin strong binding [10], [16]. When regulated by caldesmon-calmodulin, dependent on the caldesmon-calmodulin activation state, smooth muscle tropomyosin is stabilized in the open or the closed state, increasing or decreasing the rate of myosin cycling compared to the rate without any tropomyosin being present [10], [17]. Tropomyosin forms chains along actin filaments by a head-to-tail overlap of consecutive tropomyosin molecules. This overlap leads to an increased cooperativity in the switching between the ON and the OFF state. Compared to striated muscle tropomyosin isoforms, a stronger cooperativity between tropomyosin displacement due to stronger end-to-end binding between tropomyosin molecules is observed, as well as a greater bias for the ON conformation [16], [18], [19]. Similar to striated muscle tropomyosin, smooth muscle tropomyosin facilitates cooperative binding of myosin to actin: above a critical ratio of myosin heads per actin monomer, myosin heads cooperatively displace tropomyosin into the ON state so that further myosin binding is facilitated; below a critical density or activation by phosphorylation, tropomyosin remains mostly in the OFF state [20], [21] and inhibits myosin cycling [10], [19].

Tropomyosin is expressed from the same two genes in non-muscle, striated muscle, and smooth muscle cells. In smooth muscle, alternative splicing yields two smooth muscle specific isoforms (tropomyosin-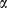 and tropomyosin-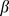), one from each gene [22]. *In vivo*, tropomyosin-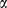 and tropomyosin-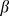 mostly occur as 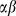 heterodimers, making functional differentiation between the isoforms difficult [10], [22]. In disease states, however, expression differences between both isoforms can be observed [6], raising the question of functional differences between these two isoforms, especially in interaction with other differentially expressed contractile protein isoforms. Crystallized N-terminal fragments of tropomyosin-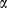 and tropomyosin-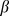 displayed differences in the heterodimerization properties of tropomyosin-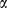 vs. tropomyosin-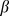 and a greater head-to-tail overlap of tropomyosin-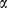 than that of tropomyosin-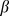 [23]. These structural results were interpreted as indication of negligible differences in tropomyosin's interface for actin binding and more important differences in the surfaces available for mediation of actin-myosin interactions as well as the binding of other proteins [23]. However, actin affinity (in terms of 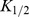 binding constants) of smooth muscle tropomyosin-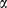 was found to be 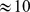 times greater than that of tropomyosin-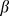 [24], [25].

**Figure 1:**
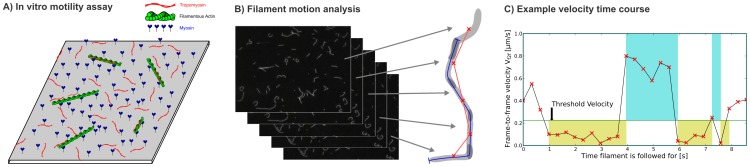
*In vitro* motility assay and video analysis. A) Purified smooth muscle myosin motors are immobilized on a microscope cover slip and propel fluorescent actin filaments in the presence of ATP. For conditions whose protein combinations contained tropomyosin (Tab. 1), tropomyosin was added into the assay buffer in excess of actin. B) Filament images are extracted from and tracked across consecutive video frames. The filament trace velocity (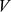) is determined from the trace resulting from the whole tracking of a filament (blue line). The frame-to-frame velocities (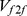) are determined from the centroid displacements between every two consecutive frames (centroids – red crosses, displacements – red lines). C) The motile fraction (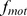), stop times (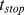
– beige regions), and run times (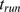
– light blue regions) are determined from 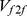 time courses.

In this study, we use an *in vitro* motility assay to investigate differences in the propulsion of “vascular” 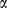-actin vs. “enteric” 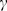-actin by smooth muscle myosin in the presence of smooth muscle tropomyosin-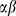, tropomyosin-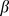, or in the absence of tropomyosin, see Fig. 1 A and Tab. 1. We develop and simulate a mathematical model to establish the differences in actin-myosin interaction kinetics that underlie the experimentally observed differences.

**Table 1:** Experimental conditions.

Protein combination	Short name	Flow-through chambers	Videos
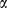 -actin	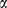 A	23	69
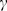 -actin	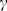 A	25	75
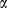 -actin and tropomyosin- 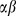	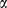 A-Tm 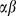	20	60
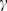 -actin and tropomyosin- 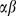	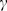 A-Tm 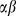	22	65
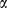 -actin and tropomyosin- 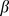	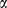 A-Tm 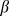	17	51
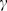 -actin and tropomyosin- 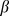	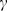 A-Tm 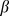	21	62

Actin and smooth muscle tropomyosin isoform combinations used in each condition, with abbreviated short name and number of experiments and videos.

## Results

### Actin length resolved features of *in vitro* motility

**Figure 2:**
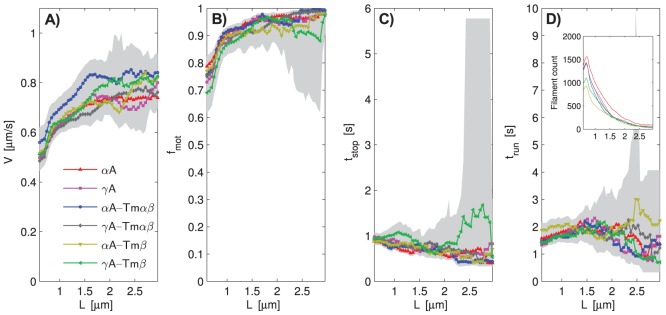
*In vitro* actin sliding features resolved by filament length. Panels A–D show the actin sliding features average sliding velocity (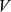), motile fraction (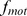), stop time (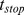), and run time (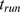), respectively. Sliding window range 0.3–3.25 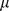
m, window width 0.59 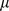
m, 50 equally spaced windows, 500 bootstrap data sets per condition, gray areas are 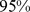 confidence intervals. Inset in panel D: number of filaments within length windows, counted separately for each protein combination. Note that 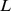 does not start at 0 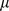
m, but at 0.6 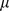
m.

Using our specifically developed analysis software, we extracted the following features of actin sliding: mean sliding velocity (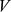), the motile fraction (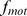), the average run time (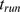), and the average stop time (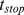) (Fig. 1 B, C). These features were extracted for the different experimental conditions (Tab. 1) and resolved by actin filament length (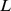) (Fig. 2). For 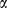
A-Tm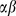 a consistent 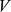 increase is apparent (Fig. 2 A). 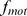, 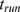, and 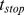 do not immediately suggest consistent differences, (Fig. 2 B–D). In spite of high filament counts (Fig. 2 D, inset), the width of the confidence intervals compared to potential differences makes a direct, conclusive inference difficult, especially for 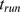 and 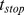 at 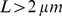.

### Baseline conditions and regulated conditions

**Figure 3:**
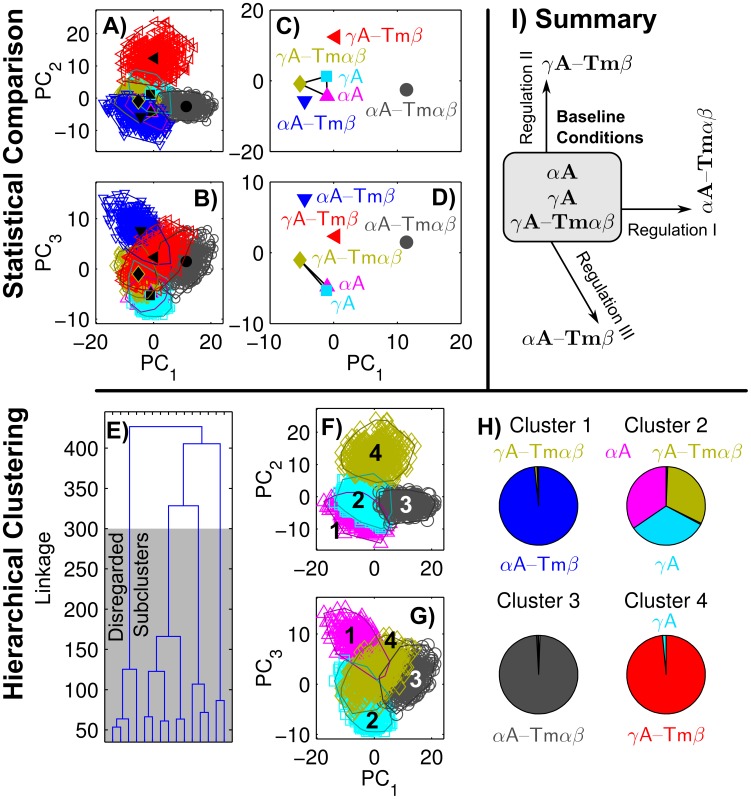
Regulation occurs in three actin and tropomyosin isoform dependent modes. A, B) For each condition (Tab. 1), the main data set (solid black symbols) and the bootstrap data sets (hollow colored symbols) demonstrate the location and variation in the first three Principal Components (PCs). A convex hull is drawn around all bootstrap data sets belonging to each condition (thin solid lines). C, D) Solid lines connect conditions that show no statistically significant differences, the absence of a connecting line indicates significant separation. E) Linkage in a tree describing agglomerative hierarchical clusters of all bootstrap data, suggesting the use of four clusters for further analysis. F, G) Unsupervised classification of bootstrap data into four clusters (represented by color and symbol shape), enclosed in convex hulls (solid lines). H) Contribution of each experimental condition to the four clusters. I) Summary scheme.

The 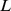 resolved features represent a simultaneous measurement of 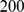 values, whose interdependence cannot be judged *a priori*. We applied a Principal Component Analysis (PCA) to reduce the dimensionality of our data and remove correlations between values, which would otherwise inflate statistical significance. Transformation into the three Principal Components (PCs) explaining most of the variance indicates that consistent differences between the experimental conditions exist (Fig. 3 A, B). Our statistical analysis detected no differences between 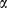
A, 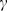
A, and 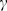
A-Tm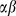, which will therefore be referred to as baseline conditions that show no effect; 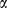
A-Tm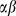, 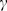
A-Tm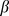, and 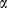
A-Tm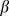 are all different from the baseline conditions, as well as from each other (Fig. 3 C, D). To support the conclusions from our statistical analysis, we executed a hierarchical cluster analysis. Based on the relatively large reduction of linkage when going from four to five clusters, a number of four clusters was chosen (Fig. 3 E). In the PC space, the four clusters appear similar to the above separation into one baseline and three regulated conditions (Fig. 3 F, G). Indeed, the four clusters form a clear representation of the 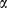
A, 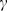
A, 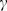
A-Tm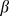 baseline conditions, and the three distinctly regulated conditions 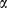
A-Tm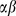, 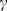
A-Tm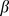, and 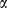
A-Tm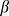, (Fig. 3 H). Thus, two independent methods of statistical assessment indicate that only 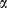
A-Tm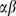, 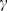
A-Tm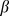, and 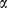
A-Tm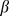 are significantly regulated, while for each of them the regulation affects actin sliding in the *in vitro* motility assay in a distinctly different manner (Fig. 3 I).

### Molecular mechanical effects of regulation

**Figure 4:**
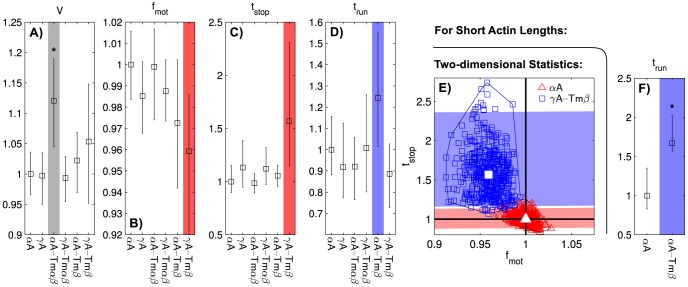
Fold changes in *in vitro* motility features. All fold changes are relative to 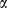
A, averaged over 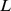, error bars are 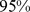 confidence intervals. A–D) Motility features averaged over whole 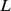 range. 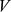 is statistically significantly elevated for 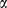
A-Tm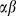. E) Statistically significant differences for 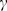
A-Tm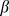 become apparent by using 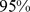 confidence bands in a two-dimensional space spanned by 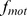 and 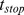 (red and blue area, projection of bootstrap data points onto vector connecting both conditions). F) 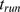 is statistically significantly elevated for 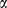
A-Tm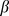 in the short 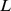 range. Windowing parameters as in Fig. 2, except for panel F: 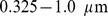, window width 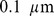, 25 windows.

Next, we wanted to attribute the differences that had been detected using PCA to molecular mechanical features. Thus, we evaluated the motility features' fold changes relative to 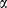
A, averaged over 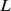. For 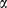
A-Tm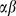, 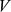 is statistically significantly increased to 1.12 times the baseline value (Fig. 4 A). For 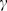
A-Tm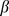, 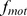 is decreased to 0.96-fold, 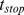 is increased by a factor of 1.6 relative to the baseline value, though both changes show up only as strong trends (Fig. 4 B, C). For 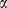
A-Tm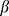, 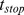 is elevated 1.3-fold, which also shows up as a strong trend only (Fig. 4 D). When 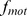 and 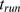 are analyzed together, the joint fold changes for 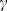
A-Tm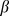 become statistically significant (Fig. 4 E). When only short actin is considered, 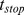 is statistically significantly elevated to 1.7 times the baseline value (Fig. 4 F). Note that each condition's differences are found in different features, which is coherent with the PCA finding that the regulated conditions are each affected by tropomyosin in a distinct manner.

### Kinetics underlying regulation

To theoretically understand the regulatory effect that tropomyosin has on actin-myosin interactions, we constructed a mathematical model of the kinetics of a myosin-coated surface interacting with actin filaments of different length 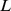. Stochastic simulations of our model produce 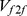 time courses (Fig. 1 C). Averaging these time courses gives 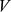, all other features of actin sliding can be extracted in exactly the same way as from experimental data. Our model is an extension of our earlier model of the group action of myosins propelling actin filaments in the *in vitro* motility assay [26]. Briefly, the model assumes that myosin moves actin by two mechanical steps, the main power stroke and a secondary mechanical step preceding myosin detachment [27], [28]. When several myosins are simultaneously bound to the same actin filament, they are mechanically coupled via the filament. Thus, the individual myosins' steps cause a change in the mechanical configuration of the overall system of bound myosins and the actin filament. Consequently, mechanical work might have to be exerted on or might be released from the actin-myosin system during the execution of an individual myosin's mechanical step. This mechanical work affects the strain-dependent rates of both mechanical transitions, the main power stroke and the secondary pre-detachment step. The overall number of myosin binding sites that are accessible on a given actin filament (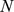) is assumed to be proportional to 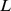. Using the helix repeat of actin (0.0355 μm) as an approximate binding site distance [27], [29], the 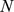 ranges were adjusted to correspond to the 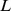 ranges used in the different analysis steps. For details regarding our mathematical model, see Text S1.

**Figure 5:**
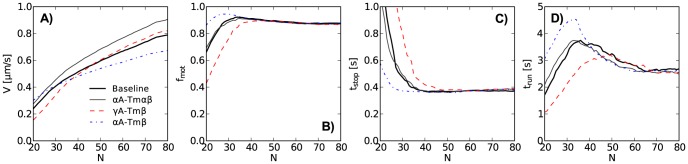
Simulated *in vitro* actin sliding features. A–D) Actin sliding features plotted vs. filament length. Motility features were extracted from simulated actin sliding in the same way as from the experimental data (Fig. 2).

**Figure 6:**
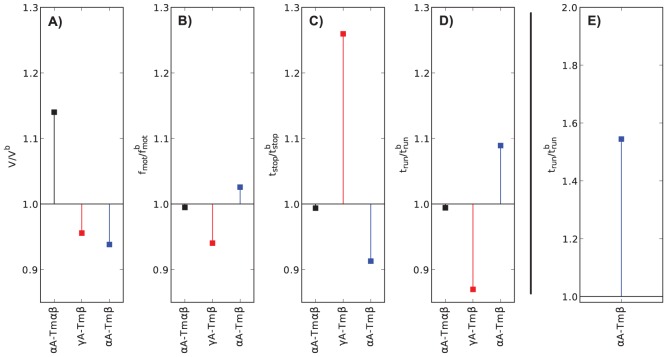
Fold changes in *in vitro* actin sliding features in model simulations. A–D) Simulated motility features averaged over whole 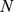 range. The fold changes were calculated in the same way as for the experimental data (Fig. 4). The altered conditions 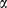
A-Tm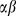, 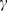
A-Tm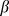, and 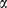
A-Tm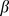 are normalized by the baseline condition (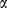
A, 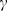
A, 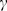
A-Tm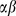). E) 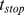 fold change for low 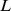 range (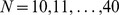).

A set of model parameters was determined to mimic the baseline condition (Fig. 5). These baseline parameters were altered so as to mimic the changes in 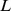 resolved features that were observed experimentally for the 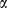
A-Tm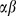, 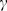
A-Tm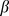, and 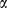
A-Tm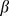 conditions (Fig. 5). The scalar fold changes in motility features were determined in the same way as from the experimental data (Fig. 6). The 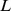 resolved motility features as well as the fold changes capture the experimentally observed differences between the baseline conditions and the conditions that exhibited statistically significant effects.

**Figure 7:**
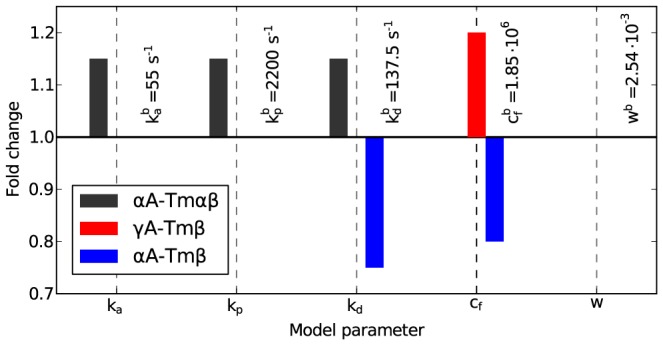
Fold changes in model parameters. The model parameters of the regulated conditions (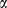
A-Tm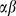, 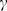
A-Tm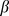, and 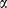
A-Tm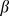) are shown, normalized by the parameters determined for the baseline condition, whose values are displayed to the right of the bars.

The changes in model parameters that were necessary to mimic the experimentally observed differences point towards the aspects of actin-myosin interaction kinetics that are changed in the different conditions (Fig. 7). For 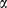
A-Tm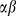, all kinetic rates (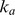, 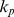, 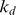) are increased 1.15-fold. For 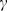
A-Tm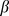, the impact of mechanical coupling between myosins on the rate of the mechanical transitions (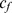) is increased by a factor of 1.2. For 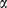
A-Tm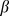, 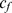 is reduced to 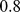 of the baseline value, and 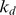 is reduced to 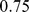 of the baseline value.

## Discussion

We investigated *in vitro* the relevance of actin and smooth muscle tropomyosin isoforms to the mechanical action of smooth muscle myosins on actin. In accordance with prior studies [4], [12]–[15], no differences between actin isoforms could be detected. However, the sequence differences between actin isoforms are confined to regions of interaction with regulatory proteins [30], suggesting potential mechano-chemical differences in the presence of such regulatory proteins. *In vitro* studies in solution (i.e. not on a motility surface) showed a different binding affinity between actin and smooth muscle tropomyosin [24], [25]. Here, we establish that, in the presence of both tropomyosin-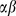 and tropomyosin-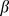, the molecular mechanics differ between 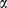
- vs. 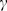-actin. Thus, the sequence differences between actin isoforms not only affect actin-tropomyosin interactions, but also actin-myosin mechano-chemistry. Importantly, we found that 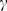-actin is significantly regulated only by tropomyosin-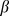, while 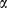-actin is regulated by both tropomyosin-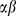 and tropomyosin-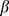.

More specifically, the regulation by tropomyosin has distinct effects on *in vitro* molecular mechanics in three regulated actin-tropomyosin combinations (experimentally determined), suggesting three different modes by which tropomyosin regulation affects actin-myosin mechano-chemistry (determined by model parameter adjustment): (1) 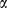
A-Tm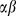 – 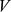 is increased 1.2-fold. This is caused by a 1.15-fold increase in the myosin attachment rate to actin, the unstrained myosin main power stroke rate, and the unstrained rate of detachment of unloaded myosin from actin. (2) 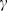
A-Tm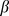 – 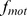 is reduced to 0.96-fold and 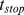 is increased 1.6-fold. This is caused by an increase in the impact that myosin-to-myosin mechanical coupling has on rates of mechanical steps of myosin by a factor of 1.2. (3) 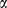
A-Tm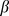 – 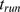 is increased 1.7-fold for short actin. This is caused by a decrease in the unstrained rate of detachment of myosin from actin to 0.75 times the baseline value and a decrease to 0.8-fold in the impact that myosin-to-myosin mechanical coupling has on rates of mechanical steps of myosin.

Note that no quantitative adjustment, e.g. minimization of sum of squared errors, was used to determine the model parameter changes stated above. In consequence, the numeric parameter changes stated above should be understood as qualitative indicators of the general nature of changes in actin-myosin interaction kinetics.

The changes in kinetic parameters determined for 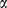
A-Tm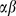 using our model-based assessment are in line with what is known for this condition from ATPase assays with skeletal muscle myosin and actin. Sobieszek determined that gizzard smooth muscle tropomyosin increases the ATPase 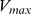, while the affinity of myosin for the actin-tropomyosin complex was not affected at myosin∶actin ratios of less than one myosin head per 4 to 6 actin monomers – which is the relevant regime for our experiment [31]. These observations were attributed to increases in the rates of the kinetic steps after myosin binding to the actin-tropomyosin complex, which is concurrent with the general increase in the unstrained kinetic rates we observed for 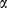
A-Tm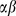. Williams et al. found results that are similar to Sobieszek's and were measured at low myosin concentrations and low ionic strengths corresponding to those used in our motility assays [18].

Sufficient evidence exists to state that smooth muscle tropomyosin does regulate smooth muscle myosin interactions with actin, and thus, the resulting molecular mechanics [10], [20], [21], [32]. Regarding the functional relevance of the smooth muscle tropomyosin isoforms, however, several not mutually exclusive mechanisms by which the isoforms could affect molecular mechanics have been put forward [22]:

1. Differences in the molecular structure of tropoymosin, in the commonly observed dimerization of tropomyosin, or in end-to-end binding of the dimers, i.e. differences attributed to tropomyosin only, not to other binding partners (high sequence and structure variation in end-to-end binding domains [23], impaired long chain formation in head-to-tail overlap region mutants [25]);
2. Differences in the location and configuration of tropomyosin dimers attaching to the actin filament surface, leading to increased or decreased blocking of other actin binding partners, i.e. differences attributed to the interaction of tropomyosin and actin (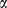
- vs. 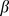-isoform lead to 10-fold differences in actin-tropomyosin dissociation constant [24], Tropomyosin-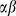 dimers exhibit specificity in their orientation when bound to actin [33]);
3. Differences that are directly attributed to the interaction between actin binding proteins and tropomyosin. (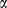
- vs. 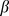-tropomyosin lead to almost two-fold difference in myosin(S1)-actin dissociation constant [24], troponin specific binding site that occurs in skeletal, but not smooth muscle, 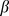-isoform of tropomyosin [25], binding between smooth muscle myosin and smooth muscle tropomyosin without actin present [34]).

With regards to smooth muscle contraction, smooth muscle myosin is the most central interaction partner of actin. We investigated its mechanical action on actin in the background of different actin and tropomyosin isoforms' interaction. Because we found that tropomyosin isoforms are indeed relevant to the regulation of actin-myosin interactions, all three mechanisms are possible for actin-tropomyosin-myosin interactions. However, the observed difference between the tropomyosin isoforms depends on the actin isoform. This suggests direct interactions between the actin filament and tropomyosin, highlighting the second mechanism.

Our mathematical model does not include tropomyosin-mediated myosin binding cooperativity. Binding cooperativity is often assessed by changing the myosin-actin ratio or the myosin activation level [10], [19]–[21]. Within the scope of this study, one detectable effect of binding cooperativity differences would be a shift in the actin length at which bifurcations between non-motile and motile behavior occur [26]. These bifurcation lengths depend on the number of myosins effectively bound to actin and would be affected by cooperativity-mediated changes in the effective rate of myosin binding to actin. We found no significant shifts in these lengths between the conditions, and therefore no indication of differences in binding cooperativity.

Like any automation of a manual analysis procedure, our video analysis software makes the analysis of large data sets feasible and prevents differences occurring between different days or operators. A specific advancement is the automated machine learning-based approach to quality control of the filament traces. Further, a result management framework was devised, which allows keyword-based queries into annotated data sets and the application of custom analysis functions. Utility functions allow the creation of customized MatLab scripts to interact with results. This supports customized analyses of existent data sets also by computational scientists without their own motility assays, as well as the “high throughput” necessary for determining statistical distributions and 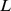 resolved curves of motility features. The MatLab scripts with instructions are released as open source (In Vitro Motility Assay Automated Analysis – ivma^3^, http://code.google.com/p/ivma3/). FIESTA is another openly accessible analysis software that can be used for *in vitro* motility assays [35]. It reaches nanometer precision and allows interactive assessment of filament motility in a graphical user interface. Differently, our software provides less precise image analysis and tracking at the benefit of fast processing of a high number of experiments and the possibility to execute specific analyses on large data sets in an automated fashion.

The statistical assessment uses bootstrapping to maintain the high filament count that is necessary for a high 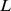 resolution while still giving account of the variation present in the experiment. To explore the results and counteract inflation of statistical significance resulting from 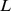 resolved analysis, PCA was used on the bootstrapped data sets. We could not find existent examples of this combination of PCA and bootstrapping – other studies estimate the variation of PCA itself [36], [37], or assess the variation of bootstrap scores (loadings) [38], [39].

More detailed assessment of *in vitro* motility and the observed specificity of regulation require more specific theoretical explanations of the molecular mechano-chemistry underlying these observations. Our relatively simple stochastic model generated data sets that were analyzed in the same way as actual experimental data, indicating how the different actin and tropomyosin isoform combinations affect actin-myosin interaction kinetics. While providing a perspective beyond mere presentation of our experimental findings, the simplicity of our model as well as the procedure by which model parameters were adjusted to mimic the experimental observations call for future work. From an experimental perspective, molecular mechanical assays using expression and site-directed mutagenesis of actin and tropomyosin seem promising.

## Materials and Methods

### Experimental methods

#### Protein purification and preparation

Contractile proteins were purified from tissues donated from the slaughterhouse as specified below. Myosin was purified from pig stomach antrum as described previously by Sobieszek [40]. 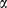-actin was purified from chicken pectoralis acetone powder as described by Pardee and Spudich [41]. 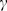-actin was purified from turkey gizzard following a previously reported protocol by Ebashi [42]. Actin was fluorescently labelled by incubation with tetramethylrhodamine isothiocyanate (TRITC P1951, Sigma)-phalloidin [43]. Tropomyosins were purified by ammonium sulfate precipitation and then collected by isoelectric precipitation at pH 4.616. Tropomyosin-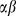 was purified from chicken gizzard, tropomyosin-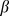 from the phasic region of pig stomach.

#### Myosin phosphorylation

Myosin (5 mg/ml) was thiophosphorylated with 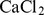 (6.75 mM), calmodulin (3.75 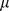
M, P2277, Sigma-Aldrich Canada), myosin light chain kinase (0.08 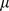
M), 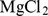 (10 mM) and ATP 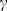-S (5 mM) by incubation with all reagents for 20 minutes at room temperature, kept at 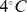 overnight, and then stored in glycerol at 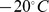.

#### *In vitro* motility assay

Flow-through chambers and buffers were prepared and used as previously described by Léguilette et al. [44]. The oxygen scavenger contained 0.16 mg/ml glucose Oxidase, 0.045 mg/ml Catalase, 5.75 mg/ml glucose. Non-functional myosin heads were removed by ultra-centrifugation of purified myosin (42,000 rpm, 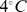, 32 min, TLA-42.2 rotor in Optima L-90K ultracentrifuge, Beckman Coulter, Fullerton, CA). In parallel with ultra-centrifugation of myosin, the buffers used to perfuse labelled actin in the regular motility assay protocol were prepared on ice to contain 0.6 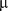
M 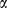
- or 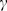-actin and, where applicable, 6 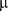
M tropomyosin-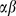 or tropomyosin-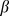 (Tab. 1). Before incubation in the flow-through chambers, myosin was diluted three-fold to 0.17 mg/ml by addition of myosin buffer. In each execution of the motility assay, 12 or 16 flow-through chambers were recorded. Batches of four flow-through chambers were incubated according to randomized conditions up until methylcellulose buffer perfusion and stored in a light-protected and humidified container. These flow-through chambers were then separately perfused with methylcellulose immediately before insertion into the microscope stage, while being preheated to 30°C during this last perfusion step (XH-2002 Small Slide Warmer, Premiere).

#### Video recording

As soon as microscope focus could be achieved, actin motility was visualized with an inverted microscope (IX70, Olympus), recorded with an image-intensified CCD camera (KP-E500, Hitachi, 30 fps), and digitized with a custom-built recording computer (Norbec Communication, Montreal, QC, Canada; Pinnacle Studio DV/AV V.9 PCI Capture Card).

### Video analysis

We developed an automated video analysis software which executes the following steps. Raw video data are preprocessed (image enhancement and frame merging to a time resolution of 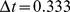 s) and turned into binary images. Filament objects and their properties are extracted from individual frames using connected components methods. Filaments are tracked throughout consecutive frames based on their centroid position and area. Frame-to-frame velocities (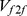) are calculated from centroid displacements between two consecutive frames. Filament length (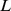) and travelled path lengths are determined based on a transformation of image objects into rectangles of same area and perimeter, the longer edge representing lengths. A filament's mean trace velocity (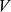) is determined by dividing the total distance that the filament's tip has travelled by the time the filament was present for (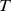). Filament traces with filament crossing events or signs of irregular motion were removed by a machine-learning algorithm, which was trained on subset of our data that we scored by hand. The automated video analysis was assessed using computer-generated mock motility videos, the automated quality control was evaluated against hand-scored data sets. For details see Text S1.

### Statistical analysis

Statistical significance was assumed for 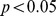. Statistical comparisons were executed by bootstrapping of the compared statistic; statistical significance was assumed where no overlap exists between the 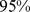 confidence intervals of the compared conditions. For details see Text S1.

## Supporting Information

Figure S1
**Brownian motion-type displacement at different time resolutions** 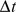 **.** A) Mean velocities expected from sliding at a constant velocity 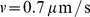 (solid line) and a velocity 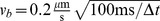 resulting from Brownian motion-type displacements (dashed line). B) Means of two Gaussians (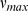 and 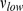) fitted to the 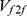 velocity distribution extracted from four videos of 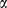-actin sliding, 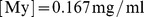. C) 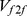 histograms for different time resolutions. The two populations that should be observable in *in vitro* motility [45] are visible only for sufficiently high 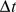, while too low 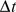 the two populations are not visually separable. Inset: computation time on a single and two processors (“cores”).(EPS)

Figure S2
**Validation of automated video analysis.** Mock videos with filaments of known 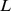 and 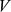 were computer-generated and subsequently analyzed. Black crosses represent input 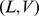 combinations, black circles detected filaments, and blue boxes indicate detected filaments for which length and velocity values were real numbers (without imaginary parts). A) Analysis at 6 frames per second, no optical noise, Brownian displacement or change of direction assumed. Only small deviations from input 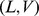 values can be observed, complex solutions occur at low 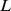. While complex solutions do not produce accurate 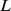 values, data points are still successfully ordered along the 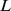 axis. B) As in A), but analysis at 3 frames per second. For high sliding velocities 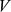, the filament length is over-estimated due to motion of the filament in frames that are merged (motion blur). C) Filaments created with fluorophore brightness fluctuations, Brownian displacement of fluorophores, and curved filament motion.(EPS)

Figure S3
**Robustness of velocity estimates to filament width.** Four 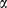-actin videos were analyzed with different Black-White thresholds (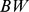). Increasing 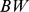 decreases filament width (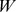) and filament length (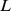). The mean sliding velocity (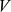) is mostly unaffected for (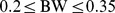). Outside this range, 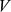 is affected as well. For 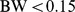 an increasing number of valid filaments is detected, while computation time increases sharply (inset). Data shown are arithmetic means with standard errors.(EPS)

Figure S4 **Assessment of automated filament rejection.** A) The predicted error rate reduces towards a plateau above 100 trees in the decision tree ensemble, indicating that 150 trees will ensure maximally achievable performance. B) Receiver Operating Characteristic (ROC) curve for cross-validation between motility data recorded on two different days. C) Cost optimization to determine acceptance threshold above which filaments are kept in the data set. Line styles: solid – overall cost of misclassification, dashed – false positive rate, dash-dotted – false negative rate. Colors: Gray – training on manual scoring from Day 1, assessment on manual scoring from Day 2; black – training on Day 2, assessment on Day 1. Cost: false positive – 5, false negative – 1. 150 trees were used in B and C. Filter for corner detection: Gaussian, parameters [21,1], 2.5, maximal number of corners: 200, parameters for corner detection: sensitivity factor 0.2, quality level 0.15.
(EPS)

Figure S5
**Assessment of Type I Error rate in the length-averaged fold change analysis.** Shown are empirical cumulative probabilities of the difference in confidence interval limits (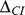) for comparing two random resamples of the baseline condition (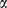
A). A Type I Error (detection of a statistically significant difference in the absence of an actual difference) is indicated by 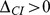. For the four features of *in vitro* motility (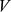, 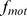, 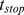, 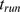), no 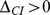 could be detected. The 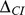 distributions are several distribution widths below 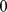, which further indicates that 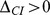 should occur very rarely. Distributions were created from 300 comparisons of resamples.(EPS)

Figure S6
**Changes in** 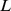 **resolved features for changing the model parameters.** Each model parameter (one per row) was changed from 0.25 to 1.75 times (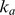, 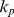, 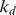) or 0.75 to 1.25 times (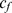, 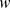) its baseline value (15 equally sized steps, indicated by solid lines shaded from black representing the lowest value to light grey representing the highest value; dashed line represents baseline). The resulting changes in the motility features 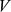, 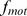, 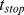, and 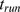 (one per column) were used to determine model parameters that mimic the experimentally observed differences.(EPS)

Text S1 **Supplementary methods.** Detailed description of Video Analysis, Statistical Analysis, and the Mathematical Model and Simulation.
(PDF)
