# Supplementary material for: Molecular Mechanical Differences between Isoforms of Contractile Actin in the Presence of Isoforms of Smooth Muscle Tropomyosin
Source: PLoS Comput Biol. 2013 Oct 24;9(10):e1003273. doi: 10.1371/journal.pcbi.1003273 (PMC3812040; doi:10.1371/journal.pcbi.1003273)
Supplement: Text S1 — Supplementary methods. Detailed description of Video Analysis, Statistical Analysis, and the Mathematical Model and Simulation. (PDF) [file pcbi.1003273.s007.pdf]

Supplementary Text S1: Supplementary Methods for Molecular Mechanical Differences between Isoforms of Contractile Actin in the Presence of Isoforms of Smooth Muscle Tropomyosin

Lennart Hilbert[1,2,3], Genevieve Bates[3], Horia N. Roman[4,3], Jenna L. Blumenthal[3], Nedjma B. Zitouni[3], Apolinary Sobieszek[5], Michael C. Mackey[1,6,2], Anne-Marie Lauzon[7,4,3,*],
1 Dept. Physiology, McGill University, Montréal, Québec, Canada
2 Centre for Applied Mathematics in Bioscience and Medicine, Montréal, Québec, Canada
3 Meakins-Christie Laboratories, McGill University, Montréal, Québec, Canada
4 Dept. Biomedical Engineering, McGill University, Montréal, Québec, Canada
5 Institute for Biomedical Aging Research, Austrian Academy of Sciences, Innsbruck, Austria
6 Dept. Physics and Dept. Mathematics, McGill University, Montréal, Québec, Canada
7 Dept. Medicine, McGill University, Montréal, Québec, Canada ∗ Corresponding author, E-mail: anne.lauzon@mcgill.ca

Video Analysis

Preprocessing of raw motility videos: Starting from raw motility videos, n consecutive frames are merged (brightness average of grey scale images). This lowers the number of frames to analyze, improves the signal to noise ratio by reducing brightness fluctuations, and reduces the influence of Brownian motion type noise (Fig. S1 A-C). In this study, every 10 frames were averaged into one, giving an effective time resolution of $\Delta t = 0.333\,\text{s}$. In each averaged frame, the brightness values are shifted by a scalar value so as to give the same brightness median across all frames to counter-act photobleaching and other lighting fluctuations. The brightness range of each video is rescaled to $[0, 1]$, so that the brightest pixel has the value 1, the darkest pixel 0.

Extraction of image objects: Each video frame is thresholded into a binary image, whose connected components are extracted (four neighbour connectivity). Objects of the size of one pixel are removed from further analysis right away.

Filament tracking: Filaments are tracked from one frame to the next based on a dissimilarity matrix $\hat{\mathbf{M}}$. For two consecutive frames ($n-1$ and n), the dissimilarity matrix is an $N_{n-1} \times N_n$ matrix, where N_n is the number of filaments in frame n. The matrix elements are

$$M_{k,l} = (A^k_{n-1} - A^l_n)^2 + (\hat{\mathbf{c}}^k_{n-1} - \hat{\mathbf{c}}^l_n)^2, \tag{1}$$

that is, the dissimilarity matrix $\hat{\mathbf{M}} = (M_{k,l})$ is determined from the area A^k_{n-1} (number of pixels times area per pixel) and weighted centroid $\hat{\mathbf{c}}^k_{n-1}$ (center of mass of grey scale pixel values) of the k-th filament in the old $(n-1)$ frame, and A^l_n and $\hat{\mathbf{c}}^k_n$ of the l-th filament in the new (n) frame. All $\hat{\mathbf{c}}$ are two-dimensional vectors, and potentiation by 2 indicates the scalar product formed by the dot product of a vector with itself (i.e. the sum of squared elements).

The properties of a filament in a specific single frame n are stored under a Metaindex m. Given that in each frame n a Metaindex m uniquely references a single filament, the Metaindices can be used for filament tracking – a filament holding the same Metaindex m in two frames is understood as the same filament tracked over several frames.

The task of tracking, formulated in the above way, is to use $\hat{\mathbf{M}}$ to assign Metaindices in a new frame n based on those from an old frame $n-1$. First, the row minimum is found for all rows of $\hat{\mathbf{M}}$. All values except these row minima are set to zero, e.g.

$$\begin{pmatrix} 1 & 3 & 4 \\ 2 & 5 & 6 \\ 3 & 2 & 4 \end{pmatrix} \rightarrow \begin{pmatrix} 1 & 0 & 0 \\ 2 & 0 & 0 \\ 0 & 2 & 0 \end{pmatrix}. \tag{2}$$

In this matrix, the three different possibilities by which a Metaindex can be assigned to a filament in the next frame are shown: (1) In the first and the second rows, the minimum is at the first position. This indicates that two Metaindices from the current frame point to the same filament in the next frame. In this case the filament in the next frame (in our example with index 1) is assigned a new Metaindex. (2) In the third row, the minimum is at the second position, so the Metaindex of the filament with index 3 in the current frame is forwarded to the filament with index 2 in the next frame. (3) The filament with index 3 in the next frame is not assigned a Metaindex from Metaindex forwarding, therefore it is assigned a new Metaindex. Whenever a filament in the next frame is not assigned a Metaindex, a new Metaindex is introduced. It is simply +1 greater than the greatest Metaindex existent at that point, which ensures uniqueness of Metaindices.

Two more checks are executed during Metaindex forwarding: A length change check and a separation check. For the length change we compare the size between two filaments that are about to be assigned the same Metaindex in two consecutive frames. We check if 1) the ratio of the lengths of the n and $n+1$ filament is greater than a defined parameter C_{rel} or smaller than $1/C_{rel}$, or 2) the lengths of the $n-1$ and the n filament differ by more than a defined parameter C_{abs}. If so, we conclude that an unreasonably big length change occurred and assign a new Metaindex for the n filament. A separation check ensures that two filaments which were chosen to be assigned the same Metaindex in consecutive frames will actually form a connected trace. If they share at least one common pixel, the trace is connected, and the check is passed. If the filaments in consecutive frames do not share any pixels, they are assumed to be different filaments and a new Metaindex is introduced for the filament in the second frame. Similar approaches to following filaments were used in earlier works [1–4]

Frame-to-frame velocity: The frame-to-frame velocity (V_{f2f}) between two consecutive frames is the centroid displacement divided by the time resolution Δt. V_{f2f} is used where time courses of filament motion are desired (Fig. 1 B). However, for longer filaments an increasing underestimation of sliding velocity occurs due to the centroid cutting corners when the filament turns [5].

Filament length and trace-based sliding velocity: Filaments and traces, both of elongated shape, are transformed into rectangles of same area (A) and perimeter (P). The object length then is the longer x^+ of the two rectangle edges [6]

$$x^{\pm} = \frac{P}{4} \pm \sqrt{\frac{P^2}{16} - A}. \tag{3}$$

The trace-based velocity (V) is calculated from the distance that a filament travelled along its trace during the time it was observed and tracked for (T). The trace is constructed by merging the filament's binary images from all frames (Fig. 1 B). The filament length (L) is determined by averaging over the filament length in all frames that the filament was observed in. L is subtracted from the trace length, resulting in the distance travelled by the filaments tip along its sliding path (Fig. 1 B). This trace length is divided by T to give V. Unlike the centroid method, V is not affected by systematic errors resulting from curved motion.

Features of actin motility: The mean sliding velocity is calculated as the mean of trace velocities V. Trace velocities are used instead of the centroid-based frame-to-frame sliding velocities (V_{f2f}) to prevent velocity under-estimation for long filaments (Fig. 1 B) [5]. The motile fraction (f_{mot}) is the fraction of V_{f2f} greater than a threshold velocity V_t (Fig. 1 C). Based on another threshold velocity V_t^t, the V_{f2f} are scored as stopped ($V_{f2f} \leq V_t^t$) or motile ($V_{f2f} > V_t^t$). Periods spent in the non-motile state without transition into the motile state are called stop times t_{stop}, conversely periods spent in the motile state without transition into the non-motile state are called run times t_{run} (Fig. 1 C). In this study, $V_t = 0.125\,\mu$m/s and $V_t^t = 0.225\,\mu$m/s were used.

Assessment of video analysis

Influence of random displacement: Filament centroids get displaced due to directed filament motion and random fluctuations, which were approximated as a Brownian-motion type influence on V_{f2f}. The mean velocity of directed sliding (ν) is independent of the time resolution Δt. The velocity from Brownian motion is proportional to $(\Delta t)^{-1/2}$ (Fig. S1 A, B). This implies that excessively lowering Δt will obscure V_{f2f} distribution features which are visible at sufficiently high Δt (Fig. S1 C). For sufficient Δt, a motile and a non-motile population in the V_{f2f} can be distinguished [7], indicating that a sufficiently large Δt was chosen to discern these features of *in vitro* motility.

Accuracy of L and V values: Computer-generated mock motility videos were used to assess the analysis's accuracy. V and L were faithfully detected above the diffraction limit. Below the diffraction limit, indicated by complex L or V (Eq. 3), V and L become quantitatively unreliable, but filaments are still qualitatively ordered by L (Fig. S2 A). At a too coarse time resolution, L is overestimated as filament motion over several consecutive frames "stretches" filament images similar to a motion blur (Fig. S2 B). L and V values are mostly insensitive to curved sliding of filaments, see Fig. S2 C and Hamelink et al. [8]. However, less of the filaments successfully pass the rejection criteria (Fig. S2 C).

L below the diffraction limit: In the rectangular transformation to determine filament and trace lengths (Eq. 3) complex solutions with an imaginary component $\neq 0$ arise for $P/4 > \sqrt{A}$. This is the case where filaments that are shorter than the diffraction limit of $\approx 0.2\mu m$ appear in the videos as approximately circular objects (Fig. S2 A-C). Below the diffraction limit filament and trace lengths are not quantitatively precise. Still, longer filaments can be expected to hold a greater number of fluorophores, increasing the brightness and in turn the optical area of the filament, resulting in a greater image object. In consequence, the real parts $P/4$ could be used to qualitatively sort affected filaments according to L, even though quantitatively accurate L could not be inferred (Fig. S2 A-C).

Relevance of filament width and digitization threshold: Based on analysis results obtained with different Black-White thresholds (BW), V and L averages depend on filament width (W) (Fig. S3). However, in a Black-White threshold range between 0.2 and 0.35, V, W, and the number of analyzed filaments seem mostly unaffected. For a low $BW < 0.15$, the computation time increases sharply, indicating that optical noise and brightness fluctuations are analyzed as objects (Fig. S3, inset). L depends on BW across all BW values. This can be understood when taking into consideration that a lower BW leads to an increase in the visual area covered by objects, giving a "blobbier" appearance. In consequence, also the head and tail tips of filaments reach out further, effectively increasing L. For BW > 0.55 the number of valid filaments drops sharply, indicating that an excessively high BW effectively removes most filaments.

Machine Learning Quality Control

Filament traces can be rendered unfit for analysis by filament crossings, filaments "swirling" around attachment points on the motility surface, lateral displacement [9], or other features that lead to inaccurate V and L estimation. Initially, such traces were removed manually, leading to acceptance (1) or rejection (0) of specific traces. Decision tree models were then trained on such manually generated data sets, to replace time-consuming manual inspection of filament traces. To make trace images more uniform and thereby easier to categorize, trace images were rotated so that all images' main axes pointed in vertical direction. The binary image data; length, width, solidity, perimeter, and area of the trace; and the number and position of corners found using MatLab's `corner` function were extracted as features. These features were used to train the decision tree models on manually scored data. Using > 100 decision trees did not lead to a further decrease in the prediction error rate, indicating ≈ 100 as the number of decision trees necessary to achieve the maximally possible classification performance (Fig. S4 A). Models used in this work were based on 150 decision trees, ensuring a sufficient number of decision trees. Receiver Operating Curves for the classification of data from one day based on training on data from another day

show that a good margin for successful classification exists (Fig. S4 B). Based on cost optimization for different data set combinations, we chose an acceptance quorum of 0.7 trees accepting a filament trace (Fig. S4 C). False positives (traces that should have been rejected) were visually inspected and showed little or no features deviating from an undisturbed, elongated shape (data not shown).

Statistical Analysis

The raw data are motility assay videos recorded for the different conditions (Tab. 1), in the following the conditions are indexed by C. In each condition, a number (V^C) of different videos were recorded, in the following the videos are indexed by $v = 1, \ldots, V^C$. Let $\mathbf{v}$ be a sequence of length V^C, whose elements each refer to one video of C. In a general case, a video can occur in $\mathbf{v}$ once, more than once, or also not at all. The meaning of $\mathbf{v}$ is to describe a data set made up of the different videos referenced in $\mathbf{v}$, e.g. $\mathbf{v} = (1, 2, \ldots, V^C)$ represents the original data set in which each video of condition C is referenced exactly once. Resolution by L is executed using a sliding window approach: an overall L range ($L \in [L_{min}, L_{max}]$) is set, then the center of a window of constant width is moved along this range at equally sized steps. At each step, all filaments from the videos contained in $\mathbf{v}$ (multiply referenced videos are included multiply) within the windows current L range are gathered, the specific motility feature is calculated for all filaments, and the arithmetic mean of these feature values is stored as the according element of a result vector ($\mathbf{R}^C_\mathbf{v}$). In this study, 50 length windows were applied for each of the four features (Fig. 2), resulting in an $\mathbf{R}^C_\mathbf{v}$ with a length of 200 elements. Within this formalism, $\mathbf{R}^C_{\mathbf{v}=(1,2,\ldots,V^C)}$ refers to the original data set with all videos contained exactly once; this can be likened to a "mean" in a scalar measurement. However, when the videos referenced by $\mathbf{v}$ are assigned by random draws with replacement, effectively bootstrap resampled $\mathbf{R}^C_\mathbf{v}$ are created, which serve as empirical distributions around the "mean".

Statistical assessment in Principal Component space: PCA was applied to reduce feature dimension and remove correlations between different elements in the $\mathbf{R}^C$ ($\mathbf{v}$ omitted from notation) values to prevent inflation of statistical significance. First, the bootstrap $\mathbf{R}^C$ from all C were pooled to execute the PCA on all bootstrap data at once. The elements of the bootstrapped $\mathbf{R}^C$ were transformed into z-scores (centered on 0 by subtraction of the mean and normalized by standard deviation in each element) and the first 3 PCs were considered in the statistical assessment (Fig. 3 A, B).

After transformation into PC space, the overlap of 95% intervals of the empirical distributions of the bootstrapped $\mathbf{R}^C$ was assessed to detect if statistically significant differences with $p < 0.05$ are present. To compare two conditions $C = I$ and $C = II$, the original (not bootstrapped) $\mathbf{R}^I$ and $\mathbf{R}^{II}$ PC coordinates were determined using the PCA transformation matrix determined based upon the bootstrapped $\mathbf{R}^C$, giving $\mathbf{r}^I$ and $\mathbf{r}^{II}$, respectively. A vector $\mathbf{d} = \mathbf{r}^{II} - \mathbf{r}^I$ connecting both $C = I, II$ was determined and the bootstrapped $\mathbf{R}^I$ and $\mathbf{R}^{II}$ were projected onto that vector using a simple scalar (dot or inner) product. Then, empirical 2.5% and 97.5% percentiles for each C were constructed from these one-dimensional values (Ward's bootstrap confidence intervals). Where no overlap between these percentiles existed, statistical significance was inferred ($p < 0.05$) (Fig. 3 C, D).

While PCA and bootstrapping are standard methods, their combined application is seen more rarely. In other studies, for example, bootstrapping is used to estimate variance in the PCA procedure itself [10,11], or a data set of several measurements is bootstrapped into mock data sets for each of which an independent PCA is executed to determine statistics of the PCA scores [12,13].

Hierarchical clustering: The bootstrapped $\mathbf{R}^C$ in PC space were pooled across all C, and then subjected to agglomerative hierarchical clustering to rediscover the conditions (Fig. 3 E-H). The Ward method and an Euclidean metric were used.

Length-averaged fold change analysis: αA was chosen as a reference condition ($C = 0$), by which the other conditions were normalized. All bootstrapped $\mathbf{R}^C$ are divided element-wise by the values of the reference condition's $\mathbf{R}^0$ original (not bootstrapped) feature vector $\mathbf{s}_C$ values. From the normalized bootstrapped $\mathbf{R}^C$, the V, f_{mot}, t_{stop}, and t_{run} are then averaged over L (arithmetic mean), resulting in scalar fold change values.

To estimate the rate of type I errors (false positive rate), the rate of rejection of the null hypothesis in absence of an actual difference was determined. We executed length-averaged fold change analyses as described above, with the difference of comparing the baseline condition (αA) to itself. Due to the bootstrapping employed in this procedure, two executions of the procedure correspond to a random reshuffling of the same data set. This preserves the same original data set, thus creating two data sets that have no difference between them but do account for the fluctuations being present in the recorded data. The rejection of the null hypothesis, i.e. the detection of statistically significant differences, applied when the confidence intervals did not overlap. This would be the case if

$$CI_{1,1} - CI_{2,2} > 0 \quad \text{or} \quad CI_{2,1} - CI_{1,2} > 0, \tag{4}$$

where $CI_{i,j}$ are the limits of the confidence intervals. $i \in \{1,2\}$ refers to the first and second resample, respectively. $j \in \{1,2\}$ refers to the smaller and greater limit of the confidence interval, respectively. In consequence, a significant difference would be detected if

$$\Delta_{CI} = \max\left(CI_{1,1} - CI_{2,2}, CI_{2,1} - CI_{1,2}\right) > 0. \tag{5}$$

Empirical distributions of Δ_{CI} indicate that, given our specific assessment method, number of measurements, and fluctuations in our data, the detection of statistically significant differences in the absence of actual differences is highly unlikely (Fig. S5).

Mathematical Model and Simulation

We use a mathematical model developed in one of our earlier studies [14]. Here, we give only the essential assumptions, expressions, and descriptions. We simulate N myosin binding sites ($N = L/0.0355\,\mu$m [15, 16]) which are mechanically coupled via the actin filament as a rigid backbone. The myosin binding sites are referred to by an index $m = 0, \dots, N$, and have a chemical state $c_m \in \{0,1,2\}$ (Pre power stroke state, Post power stroke state, unbound state) and a mechanical displacement from their unstrained position $x_m \in \mathbb{R}$ (in units of μm). Based on this, σ_m describes if a binding site m currently has a myosin bound to it:

$$\sigma_m = \begin{cases} 0 & \text{if} \quad c_m = 0, \\ 1 & \text{if} \quad c_m \in \{1,2\}. \end{cases} \tag{6}$$

Further, the unstrained position of a myosin bound to binding site m is shifted by mechanical steps executed by the myosin. The current step length Δ_m^{step} is

$$\Delta_m^{step} = \begin{cases} 0\,\text{nm} & \text{if} \quad c_m \in \{0,1\}, \\ 4\,\text{nm} & \text{if} \quad c_m = 2. \end{cases} \tag{7}$$

This means that only the main power stroke of 4 nm length affects the equilibrium position, while the secondary power stroke of 2 nm is followed by an immediate detachment (ATP-saturated buffer) [17].

The kinetics of the individual binding sites consist of a unidirectional reaction cycle ($0 \to 1 \to 2 \to 0$), with transition rates k_a (attachment, $0 \to 1$), k_p (main power stroke, $1 \to 2$), and k_d (detachment, $2 \to 0$) [17].

Due to actin acting as a rigid backbone, the x_m result from an interaction between all myosins currently bound to actin ($\sigma_m = 1$). The length travelled by the actin filament (a, in units of μm) also results from this mechanical interaction. For a binding site going from $c_m = 0$ to $c_m = 1$, an unstrained position

$$x_m^0 \sim \mathcal{N}(a, w) \tag{8}$$

is assigned, which is a random variable drawn from a normal distribution around the mean a (attachment without any strain) with a standard deviation of w.

The force exerted by myosin on an individual binding site m is

$$f_m = -\sigma_m x_m = -\sigma_m K(a - x_m^0 - \Delta_m^{step}). \tag{9}$$

K is a spring constant, we assume linear elasticity. After each kinetic transition, we assume that an instantaneous force equilibrium between all attached myosins is attained,

$$\sum_{m=1}^{N} f_m = 0 \Rightarrow a = \left[\sum \sigma_m (x_m^0 + \Delta_m^{step})\right] / n_{att},\ n_{att} = \sum \sigma_m. \tag{10}$$

a is left unchanged if $n_{att} = 0$.

Transition rate calculation: The current chemical state c_m as well as the work that has to be exerted for the next kinetic transition (ΔW_m) determine the rate at which a binding site m will undergo the next transition, r_m:

$$r_m = \begin{cases} k_a & \text{if} \quad c_m = 0, \\ k_p^0 e^{-\frac{2}{K} c_f \Delta W_m^{1\to 2}} & \text{if} \quad c_m = 1, \\ k_d^0 e^{-\frac{2}{K} c_f \Delta W_m^{2\to 0}} & \text{if} \quad c_m = 2. \end{cases} \tag{11}$$

$2c_f/K$ is a coefficient quantifying how strongly mechanical work exerted during a mechanical transition affects the rate of this mechanical transition. When a, x_m^0 and c_m are known, the overall mechanical work stored in the actin-myosin system can be calculated:

$$W = \sum_{\overline{m}=1}^{N} \sigma_{\overline{m}} \frac{K}{2} \left(a - x_{\overline{m}}^0 - \Delta_{\overline{m}}^{step}\right)^2. \tag{12}$$

$\overline{m}$ denotes the general summation over all myosin binding sites, irrespective of the current value of m.

We now assume that a is continuously changed to the system's mechanical equilibrium position throughout the execution of a mechanical step. It follows that

$$\Delta W_m = W_m^{after} - W_m^{before} \tag{13}$$

can be calculated from the work before (W_m^{before}) and after (W_m^{after}) a mechanical step of size d occurring at binding site m,

$$\Delta W_m = \frac{K}{2} \left\{ \sum_{\overline{m}} \left[\left(\frac{d}{n_{att}}\right)^2 + (a - x_{\overline{m}}^0 - \Delta_{\overline{m}}^{step}) \frac{2d}{n_{att}} \right] + d^2 - \left(a + \frac{d}{n_{att}} - \Delta_m^{step}\right) 2d + x_m^0 2d \right\}. \tag{14}$$

The simulation is initialized with $a = 0$, $x_m = 0$, and randomly assigned c_m. Filament motion is simulated by iteration of the following steps.

1. Instantaneous force equilibration by adjustment of a.

2. Current transition rates r_m are calculated based on the system's current state.

3. The next transition time and the binding site undergoing a transition is stochastically drawn based on a Gillespie scheme [18]. The according binding site is advanced by one step in the reaction cycle.

This sequence is repeated until either a maximal number of kinetic transitions per binding site (n_{steps}) or a maximal simulation time (t_{max}) is reached. The iteration results in a sequence of travelled lengths a, and elapsed times t. In the actual video analysis from the motility assay, these exact time courses are not accessible, instead only the difference in travelled length at time intervals Δt (time between consecutive frames in our videos) can be assessed. Accordingly, V_{f2f} were extracted from the stochastic simulation using an imposed time resolution of Δt.

Actin sliding was simulated for $N = 5, 6, \ldots, 90$. At each N, actin sliding was simulated till 100 s of simulation time or $100,000 \cdot N$ chemical transitions were reached. Features of actin motility were extracted for each N, followed by a running window analysis (Start: $N = 10$, end: $N = 90$, window width of 20). Considering a binding site distance of ≈ 0.035 μm [15, 16], this approximates the L range we analyzed in our experiments.

Correction for non-specific binding to motility surface: When this model was simulated, above $N \approx 25$, t_{run} and t_{stop} could not be measured due to V_{f2f} not falling below the threshold for determining t_{run} and t_{stop}. Occasional non-specific stopping events occur for longer filaments [14]. We introduce these stopping events by inserting Poisson-distributed $V_{f2f} = 0$ μm/s values into the V_{f2f} time trace:

$$\Delta T = -\overline{T} \log r, \tag{15}$$

where ΔT is the time between two consecutive stop events, r a random number drawn from a uniform distribution $r \in [0, 1]$. $\overline{T}$ is the mean time between two consecutive stop events,

$$\overline{T} = \begin{cases} \infty & \text{if} \quad N \leq 30, \\ \frac{20\,\mathrm{s}}{N-30} + 2\,\mathrm{s} & \text{if} \quad N > 30, \end{cases} \tag{16}$$

which has been adjusted to limit t_{run} as observed in our experiments (Fig. 2 D).

Parameter adjustment: The baseline model parameters (Fig. 7) were taken from the literature and then adjusted to fit the L resolved V and f_{mot} (k_a [19, 20], k_p [17], k_d [17], an additional factor of 5.5 was used to account for higher temperature in our experiment [14]), or estimated from L resolved V and f_{mot}, starting from values determined in our earlier study of skeletal muscle myosin [14] (c_f, w). The altered parameters mimicking the αA-Tm$\alpha\beta$, γA-Tmβ, and αA-Tmβ conditions were estimated based on Fig. S6 and comparison with L resolved features and scalar fold changes in the conditions with statistically significant differences in the experiment (Fig. 2 and 3, respectively).

References

1. Homsher E, Wang F, Sellers J (1992) Factors affecting movement of F-actin filaments propelled by skeletal muscle heavy meromyosin. Am J Physiol 262: C714–23.

2. Mansson A, Tagerud S (2003) Multivariate statistics in analysis of data from the in vitro motility assay. Analyt Biochem 314: 281–93.

3. Ruhnow F, Zwicker D, Diez S (2011) Tracking single particles and elongated filaments with nanometer precision. Biophys J 100: 2820–8.

4. Marston S, Fraser I, Bing W, Roper G (1996) A simple method for automatic tracking of actin filaments in the motility assay. J Muscle Res Cell Motil 17: 497–506.

5. Work S, Warshaw D (1992) Computer-assisted tracking of actin filament motility. Analyt Biochem 202: 275–85.

6. Root D, Wang K (1994) Calmodulin-Sensitive Interactions of Human Nebulin Fragments with Actin and Myosin. Biochem 33: 12581–91.

7. Marston S (2003) Random walks with thin filaments: application of in vitro motility assay to the study of actomyosin regulation. J Muscle Res Cell Motil 24: 149–56.

8. Hamelink W, Zegers J, Treijtel B, Blange T (1999) Path Reconstruction as a Tool for Actin Filament Speed Determination in the in Vitro Motility Assay. Analyt Biochem 273: 12–9.

9. Uyeda T, Kron S, Spudich J (1990) Myosin step size estimation from slow sliding movement of actin over low densities of heavy meromyosin. J Mol Biol 214: 699–710.

10. Timmerman M, Kiers H, Smilde A (2007) Estimating confidence intervals for principal component loadings: A comparison between the bootstrap and asymptotic results. Brit J Math Stat Psychol 60: 295–314.

11. Yua C, Quinn J, Dufournaud C, Harrington J, Rogers P, et al. (1998) Effective dimensionality of environmental indicators: a principal component analysis with bootstrap confidence intervals. J Environ Manag 53: 101–19.

12. Catlin-Groves C, Kirkhope C, Goodenough A, Stafford R (2009) Use of confidence radii to visualise significant differences in principal components analysis: Application to mammal assemblages at locations with different disturbance levels. Ecol Informatics 4: 147–51.

13. Peng Q, Zhao J, Xue F (2010) PCA-based bootstrap confidence interval tests for gene-disease association involving multiple SNPs. BMC Genet 11: 6.

14. Hilbert L, Cumarasamy S, Zitouni N, Mackey M, Lauzon AM (2013) The Kinetics of Mechanically Coupled Myosins Exhibit Group Size-Dependent Regimes. Biophys J .

15. Steffen W, Smith D, Simmons R, Sleep J (2001) Mapping the actin filament with myosin. Proc Natl Acad Sci USA 98: 14949–54.

16. Capitanio M, Canepari M, Cacciafesta P, Lombardi V, Cicchi R, et al. (2006) Two independent mechanical events in the interaction cycle of skeletal muscle myosin with actin. Proc Natl Acad Sci USA 103: 87–92.

17. Veigel C, Molloy J, Schmitz S, Kendrick-Jones J (2003) Load-dependent kinetics of force production by smooth muscle myosin measured with optical tweezers. Nat Cell Biol 5: 980-6.

18. Gillesple D (1977) Exact stochastic simulation of coupled chemical reactions. J Phys Chem 81.

19. Brenner B, Eisenberg E (1986) Rate of force generation in muscle: correlation with actomyosin ATPase activity in solution. Proc Natl Acad Sci USA 83: 3542–6.

20. Goldman Y, Hibberd M, Trentham D (1984) Initiation of Active Contraction by Photogeneration of Adenosine-5-Triphosphate in Rabbit Psoas Muscle Fibres. J Physiol 354: 605–24.
